# Herbal formula Xian-Fang-Huo-Ming-Yin regulates differentiation of lymphocytes and production of pro-inflammatory cytokines in collagen-induced arthritis mice

**DOI:** 10.1186/s12906-016-1526-x

**Published:** 2017-01-05

**Authors:** Jinyu Li, Yi Wei, Xue Li, Dashuai Zhu, Bo Nie, Jingwei Zhou, Lixia Lou, Bin Dong, Aiming Wu, Yongzhe Che, Meng Chen, Lingqun Zhu, Mingwei Mu, Limin Chai

**Affiliations:** 1 Key Laboratory of Chinese Internal Medicine of Ministry of Education and Beijing, Dongzhimen Hospital, Beijing University of Chinese Medicine, Haiyuncang Hutong No.5, , Dongcheng District Beijing, China; 2 Department of Anatomy, School of Medicine, Nankai University, Tianjin, China; 3 Department of Rheumatology, Dongzhimen Hospital, Beijing University of Chinese Medicine, Beijing, China; 4 School of Preclinical Medicine, Beijing University of Chinese Medicine, Beijing, China; 5 Department of orthopedics Rheumatology, Dongzhimen Hospital, Beijing University of Chinese Medicine, Beijing, China

**Keywords:** Xian-fang-huo-ming-Yin (XFHM), Lymphocyte, Pro-inflammatory cytokine, Nuclear factor κB (NF-κB), Janus-activated kinase-signal transducer and activator of transcription (JAK/STAT), Collagen-induced arthritis (CIA)

## Abstract

**Background:**

Xian-Fang-Huo-Ming-Yin (XFHM), a traditional herbal formula, has been used to treat sores and carbuncles for hundreds of years in Asia. Nowadays, its clinical effects in treatment of rheumatoid arthritis (RA) have been validated. In this study, we want to study its possible molecular mechanisms of regulating the differentiation of lymphocytes and production of pro-inflammatory cytokines in collagen-induced arthritis (CIA) mice for RA treatment.

**Methods:**

A high performance liquid chromatography-electrospray ionization/mass spectrometer (HPLC-ESI/MS^n^) system was used to analyze the constituents of XFHM granules. An arthritics mouse model was induced by collagen and leflunomide (LEF) was used as a positive control medicine. Pathological changes at the metatarsophalangeal joint were studied through Safranin O and immunohistochemical staining. The differentiation of T, B and NK cells was examined by flow cytometry and pro-inflammatory cytokines were assayed using an Inflammation Antibody Array assay. The expression of key molecules of the nuclear factor κB (NF-κB) and Janus kinase/signal transducers and activators of transcription (JAK/STAT) signaling pathways in spleen were studied by western-blot analysis.

**Results:**

In our study. 21 different dominant chemical constituents were identified in XFHM. Treatment with XFHM suppressed the pathological changes in arthrosis of CIA. Additionally, XFHM down-regulated the proliferation and differentiation of CD3^+^ T cells and CD3^−^CD19^+^ B cells significantly. However, XFHM had no significant effect on CD3^−^NK1.1^+^ NK cells. Further study showed that the production of pro-inflammatory cytokines had been suppressed by inhibiting the activation of NF-κB and JAK/STAT signaling.

**Conclusions:**

XFHM can regulate and maintain the immunologic balance of lymphocytic immunity and inhibit the production of pro-inflammatory cytokines, thus suppressing the pathological changes of RA. Therefore, XFHM may be used as an application of traditional medicine against RA in modern complementary and alternative therapeutics.

**Electronic supplementary material:**

The online version of this article (doi:10.1186/s12906-016-1526-x) contains supplementary material, which is available to authorized users.

## Background

Rheumatoid arthritis (RA) is a chronic inflammatory autoimmune disease with articular and systemic effects [1]. The pathogenesis of RA is not fully understood. Various inflammatory cells, including innate immune cells (mast cells, macrophages, dendritic cells and natural killer (NK) cells) and adaptive immune cells (T and B cells), are activated in RA joints, contributing to the pathogenesis of RA. Endothelial cells and fibroblast-like synoviocytes also participate in the disease progress of RA [2]. These cells constitute the synovial lining layer and participate in an inflammatory cascade, eventually inducing the destruction of cartilage and bone [3].

It is well known that RA is a T-cell dependent autoimmune disease. CD3^+^ T cells, cooperating with the inflammatory cytokines excreted by T cells, induce synovial inflammation, further leading to the destruction of articular cartilage [4]. B cells also contribute to RA pathogenesis by antigen presentation and inflammatory cytokines production [5]. Cell-cell interaction of T and B cells promote the excretion of cytokines and chemokines, reinforce the interaction of the feedback loop among T cells, macrophages and B cells, and maintain the progress of synovial inflammation and destruction of cartilage and bone [6]. NK cells have been reported to play an important role in the tissue pathology of RA [7]. NK cells have a disease-promoting function on RA tissue. A subset of NK cells expanding in inflamed synovium has been demonstrated, after which the production of interferon (IFN) γ is induced to a significant degree [8, 9]. NK cells cooperating with other cell types by cytokines and chemokines may be a potential risk for RA pathogenesis.

Pro-inflammatory cytokines play key roles in the pathophysiology of RA. It is well established that tumor necrosis factor (TNF) α and interleukin (IL)-6 play the dominant roles [10, 11]. IL-1, IL-17 and IFNγ also contribute to the process of RA [10, 12]. These pro-inflammatory cytokines mediate cell migration and cause synovial inflammation, which eventually result in cartilage and bone destruction. Nuclear factor κB (NF-κB) plays a central role in the differentiation, activation and survival of mammalian cells, contributing to RA in multiple ways. It can induce the production of pro-inflammatory cytokines and other mediators of inflammation, thereby accelerating the progress of RA pathology [13]. In addition, the Janus kinase/signal transducers and activators of transcription (JAK/STAT) signaling cascade activated by IL-6 participates in the pathogenesis of RA [14].

Xian-Fang-Huo-Ming-Yin (XFHM), is a formula described in the ancient Chinese herbal treatise *Jiaozhu Furen Liangfang*, which was compiled by Xue Ji during the Ming dynasty of ancient China. This typical traditional Chinese medical (TCM) formula has been used for hundreds of years. It is used to treat many diseases such as sores and carbuncles. We used an optimized formula of XFHM (other used name Lijie capsule) as a TCM treatment for RA and found a good therapeutic effect [15]. Additionally, previous studies from our laboratory suggested that the optimized formula of XFHM has inhibitory effects on apoptosis of lymphocytes in rats with adjuvant arthritis [16]. In previous studies, we discovered that chlorogenic acid and Luteolin, the main components of *Caulis Lonicerae Japonicae* (the monarch drug in XFHM), can inhibit the inflammatory proliferation of rat synovial cells induced by IL-1β [17] and IL-6 [18]. In addition, several studies have indicated that both the crude herbs and the active ingredients of these herbs have beneficial effects on RA. These effective properties include anti-inflammation [19, 20], anti-oxidation [21], anti-proliferation [22], promoting bone metabolism[23] and stimulating osteoblasts proliferation [24]. Therefore, we suggest that XFHM has inhibitory effects on the inflammatory proliferation of synoviocytes and the subsequent destruction of cartilage and bone.

In this study, full ingredient granules of XFHM were used as the treatment drug. The quantity control of the full composition granules of XFHM was assayed using the infrared fingerprint spectrum (IRFP) technique [25]. High performance liquid chromatography-electrospray ionization/mass spectrometer (HPLC-ESI/MS^n^) analysis was used to characterize the phytochemicals of XFHM. Leflunomide (LEF), a disease-modifying anti-rheumatic drug (DMARD), was used as a positive control medicine. Collagen-induced arthritis (CIA) in DBA1/J mice induced by immunization with bovine CII in freund’s complete adjuvant (CFA) was used as an animal model. This investigation was undertaken to determine the regulatory effects of XFHM on the proliferation and differentiation of T, B, and NK cells, and the production of pro-inflammatory cytokines in CIA mice.

## Methods

### Herb materials and preparation of XFHM

The modified formula of XFHM was composed of 12 medicinal herbs. Full composition granules of the 12 herbs were provided by Beijing Tcmages Pharmaceutical Co. LTD (Beijing, China). Quality control of the XFHM granules was executed through infrared spectrum fingerprint (IFRP). The IRFP graph is shown in Additional file 1: Figure S1. The constitution ratio of 12 herbs was *Atractylodes lancea* (2), *Ligusticum chuanxiong Hort* (2), *Paeonia veitchii Lynch* (2), *Tail of Radix Angelicae sinensis* (2), *Angelica dahurica* (3), *Radix Saposhnikoviae* (3), *Boswellia carteri Birdw* (1), *Commiphora myrrha* (1)*, Astragalus membranaceus* (8)*, Caulis Lonicerae Japonicae *(6)*, Gentiana macrophylla Pall *(3) and *Rehmannia glutinosa Libosch* (3).

### HPLC-ESI/MS^n^ analysis

HPLC-ESI/MS^n^ analysis was performed on a Shimadzu 20LC (Kyoto, Japan) coupled to a diode array detector and TripleTOF 4600+ CDS mass spectrometer (AB Sciex, MA, USA). The chromatographic separations were carried out on an Agilent Poroshell C18 (2.1 mm × 100 mm, 2.7 μm). The mobile phase consisted of a combination of A (0.5‰ formic acid and 2 mM acetic acid) and B (0.5‰ formic acid and 2 mM acetic acid in acetonitrile methyl alcohol (1:1)) with a linear gradient, 0–10 min (5–20%, B), 10–22 min (20–95%, B). The flow rate was 0.4 mL/min, the sample injection volume was 5 μl and the column and sample temperature were BOTH 40 °C. The diode array detector (DAD) was set at 200, 220, 250 and 280 nm for the real-time monitoring of the peak intensity. Mass spectra were simultaneously acquired using electrospray ionization in the positive and negative ionization (POS and NEG) modes at fragmentation voltages (40 Psi) over the range of m/z 50–1250. The data was acquired with IDA (information dependent acquisition) method and analyzed by Peak View Software™ 2.2 (SCIEX, Foster City, CA, USA).

### CIA induction in DAB1/J mice

DBA1/J male mice (7 to 8 weeks old) purchased from HFK Bioscience Co. Ltd. (Beijing, China) were immunized intradermally at the base of the tail with 150 μg of bovine type II collagen (CII) (Sigma, St. Louis, MO, USA) emulsified with an equal volume of complete Freund’s adjuvant (CFA) (Sigma, St. Louis, MO, USA). The DBA1/J mice were boosted 21 days after immunization by intradermal injection with 150 μg of CII emulsified with incomplete Freund’s adjuvant (IFA). Animal care and use were in accordance with institutional guidelines, and all animal experiments were approved by the Institutional Animal Care and Use Committee of the National Institute of state Scientific and Technological Commission.

### Drug treatment

Mice were randomly divided into 4 groups as follows (*n* = 6 per group): normal group, fed with control diet and orally administrated sterile saline; model group, fed same as the normal group; LEF (Batch No. 130126, Cinkate Corporation, Beijing, China) group, LEF, fed with control diet and orally daily administrated 2 mg/kg LEF daily for 28 days; and XFHM group, fed with control diet and orally administrated 5.3 g/kg XFHM daily for 28 days. Drug treatment started from the first day after booster immunization. Mice were treated with 0.1 mL drug solution by gavage every day. Mice were sacrificed on day 29 after treatment. Mice were anesthetized by isoflurane anesthesia (2–3% isoflurane with oxygen supply). The sample size calculation was based on the basic principles of hypothetical randomized controlled trial [26]. Peripheral blood (PB) was obtained by removing the eyeballs prior to sacrifice during anesthesia, and the left legs and hind paws and spleens were removed after sacrifice.

### Arthritic severity scores of CIA

Arthritic severity scores of CIA were monitored every 7 days after booster immunization. The arthritic severity scores for paws were used to reflect the severity of arthritis. Scores were classified as 0 (normal joints), 1 (swelling in 1 digit or joint inflammation), 2 (swelling in 2 or 3 digits or slight paw swelling), 3 (swelling in more than 4 digits and moderate swelling in the entire paw), 4 (severe swelling and deformation of the paw). Each paw was graded, and the four scores were added together so that the maximum possible score was 16 per mouse [27, 28].

### Safranin O and immunohistochemical staining

The left hind legs and paws of the mice were removed, fixed with 4% paraformaldehyde in PBS, decalcified for 10 days with EDTA, embedded in paraffin, and sectioned at 5μm thickness. For Safranin O staining, sections were placed in hematoxylin for 2 min and then washed in water for 5 min. the sections were then placed in 0.1% Safranin O solution for a further 2 min and washed again in water for 30 s. Finally, the sections were passed through a series of industrial methylated spirit concentrations (70 to 100%) for 2 min at each concentration. The sections were then clarified in xylene for 2 min. the sections were then viewed, and images were taken using a light microscope.

Immunohistochemical staining for IL-1β and IL-17 was performed. After the sections were de-paraffinized, rehydrated and washed, the sections were then antigen-retrieved with pepsin and incubated with 0.3% hydrogen peroxidase for 20 min to block endogenous peroxidase activity, followed by processing with serum for 30 min to block non-specific ligations. The sections were then treated with rabbit anti-IL-1β and IL-17 (1:100, Santa Cruz Biotechnology, CA, USA) primary antibodies overnight at 4 °C, washed and incubated with reagents from an immunohistochemical kit (Zhongshan Biotechnology Ltd, Beijing, China) in compliance with the manufacturer’s instructions and visualized with 3,3-diaminobenzidine tetrahydrochloride (DAB). Finally, the sections were counterstained with hematoxylin. The sections were then viewed and images were taken using a light microscope. The IOD (integrated optical density) analysis was performed by Image-Pro Plus 6.0 (Media Cybernetics, MD, USA). Three samples in each group were reviewed five sections. The histological changes were assessed by two experienced pathologists who were blind to the treatment.

### Flow cytometry (FACS) analysis

PB (0.2 mL) and half of the spleen were harvested after the mice anesthetized. Single-cell suspensions from CIA mouse spleens and PB were isolated. For surface marker staining, fluorescence conjugated anti-mouse-CD3e-PE, anti-mouse-CD19-PE-Cyanine7 and anti-mouse- NK1.1-FITC antibodies (CST, Boston, MA, USA) were used. FACS was performed using a FACS Calibur cytometer and analyzed using the CellQuest software (Beckman Coulter, Fullerton, CA, USA).

### Inflammation antibody array assay

PB (0.6 mL) of the mice was harvested. Sera were separated through centrifugation at 1000 g/25 min. The levels of inflammatory antibodies in sera were detected by RayBiotech® Mouse Inflammation Antibody Array 1 (AAM-INF-G1, Ray Biotech Inc. Norcross, GA, USA), according to the manufacturer’s instructions. Briefly, 50 μL of serum was used in inflammation cytokines assays. The standard array matrix consisted of an 11 × 8 dot grid on a 20 mm × 30 mm nitrocellulose membrane with 40 unique capture antibodies. The array kit included a biotinylated-antibody solution and chemiluminescent substrate. The cytokine array membrane was incubated with 50 μL of serum for two hours, and the membrane was then washed three times with washing buffer 1 for 5 min each, followed by washing buffer II for 5 min each. Cytokines were detected using the cytokine antibody for one hour, followed by HRP-labeled streptavidin incubation for one hour. Fluorescence signals were scanned by a Microarray Scanner (GenePix 4000B, Axon, SFO, CO, USA) at 532 nm. Data acquisition and analysis were executed using the data analysis software AAM-INF-G1.

### Western blot analysis

The other half of the spleens of CIA mouse was homogenized in 1 ml of a lysis buffer (Sigma, CA, USA). The extracts were cleared by spinning at 10,000 g at 4 °C for 15 min, and then diluted with the lysis buffer to achieve approximately 2 mg/ml protein concentration. Protein samples were separated on 10% sodium dodecyl sulfate (SDS)-polyacrylamide gel electrophoresis (PAGE) and transferred onto nitrocellulose membranes (Amersham Pharmacia Biotech, Uppsala, Sweden). The membranes were incubated with primary antibodies, including anti- NF-κB kinase inhibitor (IKK)α/β, anti-NF-κB p50, anti- glycoprotein (gp)-130, anti-JAK1 and anti-STAT3 rabbit anti-mouse monoclonal antibodies (CST, Boston, MA, USA), and were then incubated with horseradish peroxidase-conjugated secondary antibody. All immunoreactive proteins were visualized with SuperSignals west Pico Chemiluminescent Substrate (Thermo Scientific, Rockford, IL, USA). Densitometry plots showing the expression of proteins were normalized to glyceraldehyde 3 - phosphate dehydrogenase (GAPDH) and expressed as fold relative to the levels in mice of the normal group.

### Statistical analysis

All data are presented as the means ± standard deviation (S.D.). The statistical analyses were performed using SPSS13.0 (SPSS Inc., Chicago, IL, USA). One-way analysis of variance (ANOVA) followed by the Tukey-Kramer test for multiple comparisons were used to compare the groups. *P* < 0.05 was considered to indicate statistical significance.

## Results

### Identification of chemical constituents in XFHM by HPLC-ESI/MS^n^

**Fig. 1:**
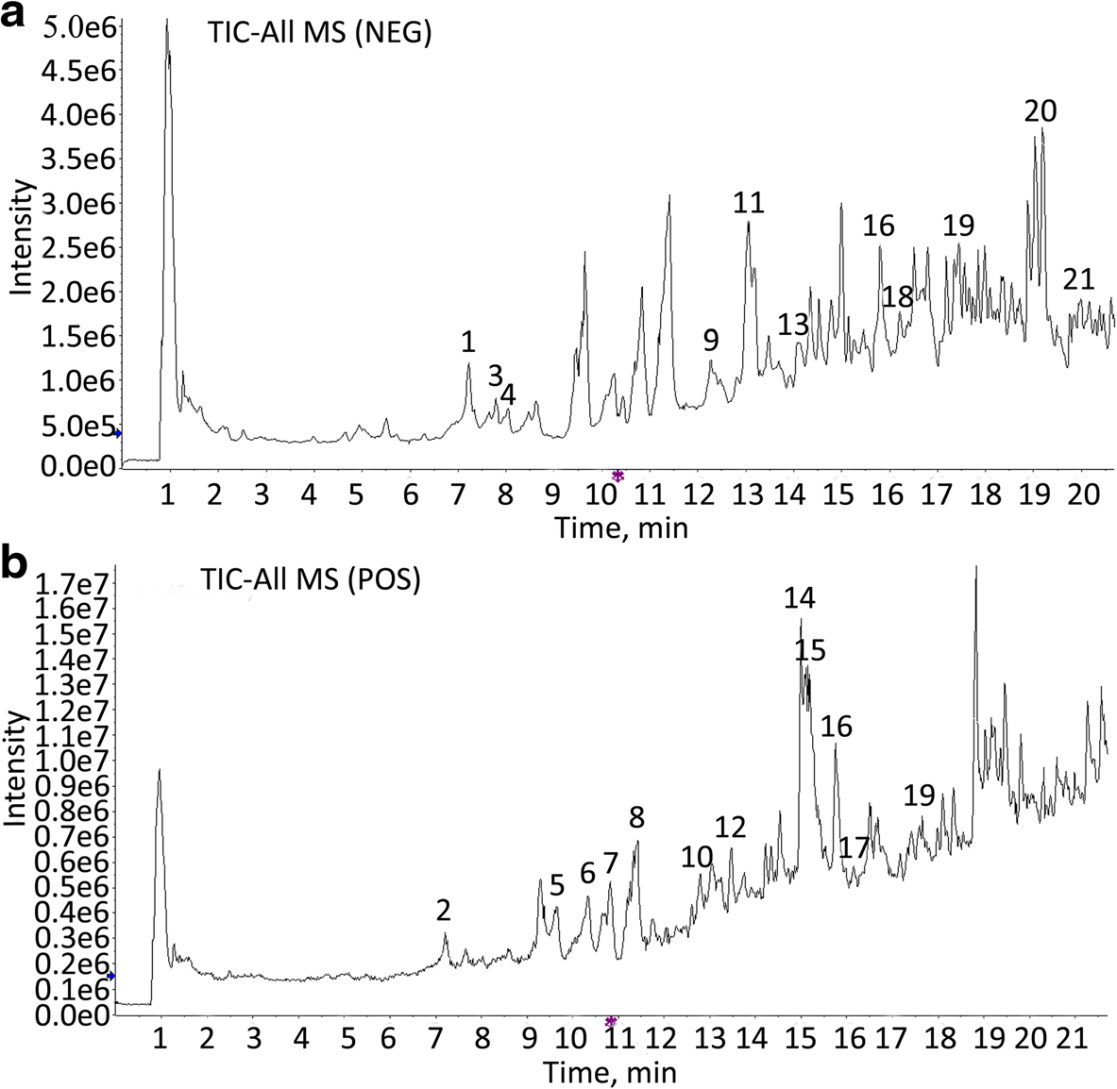
HPLC-ESI/MS^n^ total ion chromatograms of XFHM processed by different methods. **a** The negative base peak MS spectrum, **b** the positive base peak MS spectrum

Representative liquid chromatography-mass spectrometry chromatograms are shown in Fig. 1. In the HPLC-ESI/MS^n^ experiment, both negative (Fig. 1a) and positive (Fig. 1b) modes were tried. Twenty-one constituents were identified by comparing the retention time with IDA method. The identified compounds are shown in Table 1.

**Table 1:** Chemical components identified from XFHM by HPLC-ESI/MSn

No.	TR (min)	[M + H]/[M-H] (m/z)	Fragments (m/z)	Formula	Identification
1	7.03	/167.03	/152.0,132.0	C8H8O4	Vanillic acid
2	7.21	375.13	321.1,169.1	C16H24O10	Loganic acid
3	7.73	/179.03	/135.0	C9H8O4	Caffeic acid
4	8.03	/353.09	/173.0,135.0	C16H18O9	Chlorogenic acid
5	9.63	357.12	195.1,121.1	C16H20O9	Gentiopicroside
6	10.16	481.17	179.1,151.1	C23H28O11	Paeoniflorin
7	10.81	391.16	179.1,149.0	C17H26O10	Loganin
8	11.35	179.07	77.0,105.1	C10H10O3	Methyl 4-hydroxycinnamate
9	12.28	/563.14	/383.1,473.1	C26H28O14	Apiin
10	12.79	469.17	307.1,261.1	C22H28O11	Prim-O-glucosylcimifugin
11	13.27	/631.17	/491.1,271.0	C30H32O15	Galloylpaeoniflorin
12	13.44	307.12	259.1,235.1	C16H18O6	Cimifugin
13	14.03	/623.20	/461.2,161.0	C29H36O15	Acteoside
14	15.01	431.13	267.07 252.04	C22H22O9	Ononin
15	15.39	463.16	301.1,167.1	C23H26O10	Lactiflorin
16	15.81	285.06/283.06	220.1, 225.1 /268.0,211.0	C16H12O5	Wogonin
17	16.14	461.11	270.1,285.1	C22H20O11	Wogonoside
18	16.2	/271.06	/151.0,119.1	C15H12O5	Naringenin
19	17.55	269.07/267.07	197.1, 226.1 /252.0,223.0	C16H12O4	Formononetin
20	19.13	/269.08	/254.1,210.1	C16H14O4	Isoimperatorin
21	20.00	/829.46	/621.40,651.41	C41H68O14	Astragaloside IV

### Effect of XFHM on histopathological changes in metatarsophalangeal joints of CIA mice

**Fig. 2:**
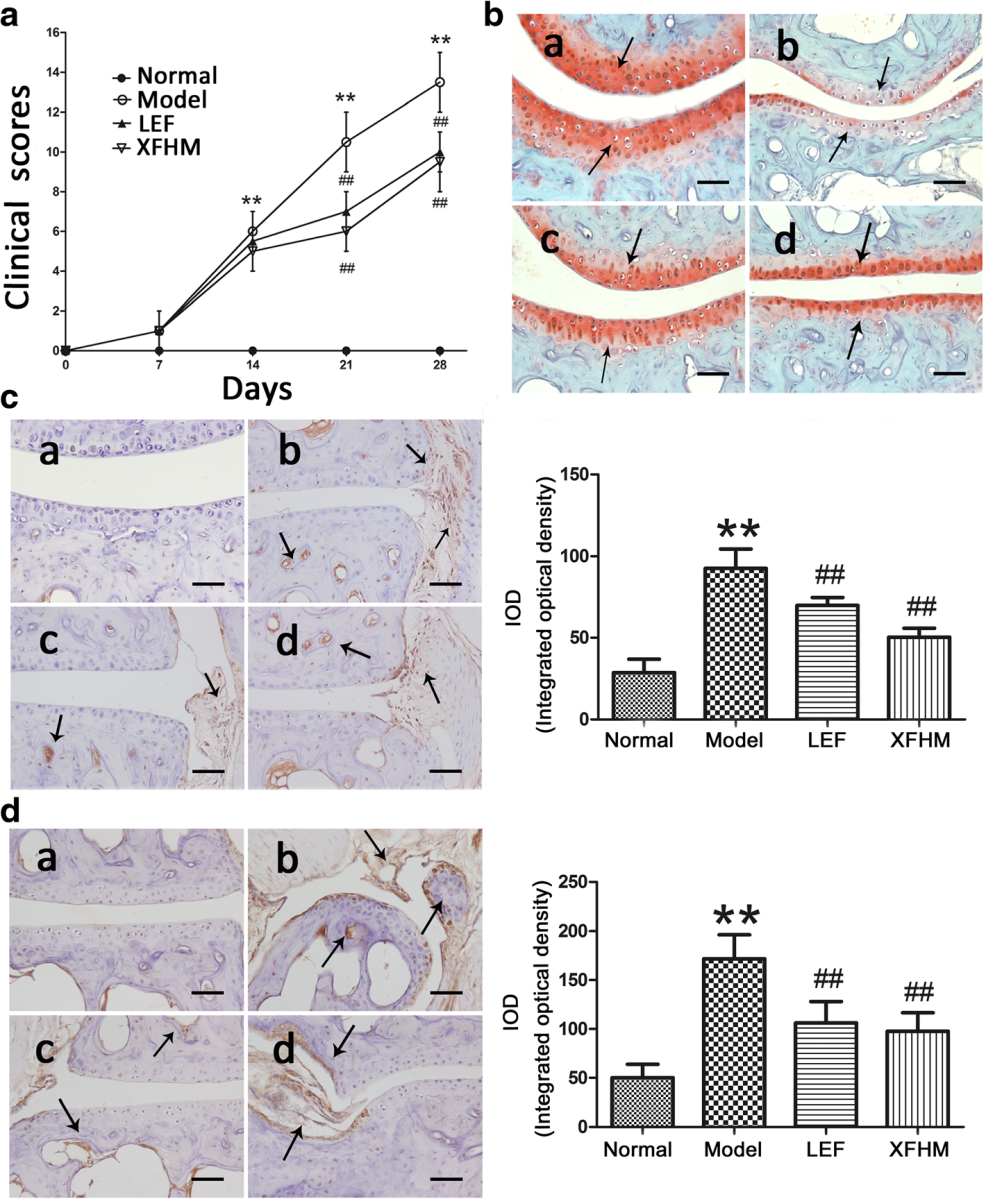
**a** Arthritic severity scores of CIA were monitored every 7 days after booster immunization. ^**^ *P* < 0.01 indicates model group vs. normal group; ^#^ *P* < 0.05 and ^##^ *P* < 0.01 indicate treatment groups vs. model group. **b** Safranin O staining of the articular cartilage. The arrows indicate Safranin O staining. **c** Immunohistochemical staining for IL-1β. The arrows indicate the location of IL-1β. **d** Immunohistochemistry staining for IL-17. The arrows indicate the location of IL-17. The results of integrated optical density (IOD) analysis are presented in the bar charts. Data are presented as the means ± S.D. (*n* = 3). The specimens were observed and photographed under a light microscope (Leica, DM RXA2) at magnification of 200 ×; a normal group, b model group, c group treated with LEF, d group treated with XFHM. The scale bar corresponds to 60 μm throughout

Mice treated with XFHM exhibited significant reductions in severity of CIA (Fig. 2a). On day 21 after booster immunization, the mean arthritic severity score for the group treated with XFHM was 6.17 ± 0.75, as opposed to 10.50 ± 1.05 for the model group. The XFHM treatment reduced arthritic severity scores at 0–28 days after booster injection in the CIA mice.

A decrease in Safranin O staining of the growth plates in CIA mice was evident prior to any morphological changes in the chondrocytes [29]. There were also differences in growth plates Safranin O staining among the groups (Fig. 2b). The intensity of Safranin O staining was significantly lower in the CIA mice relative to normal mice. After treatment with LEF and XFHM, the intensity of staining increased. Immunohistochemical detection and localization showed that IL-1β and IL-17 were expressed in the synovium, pannus and chondrocytes. As showed in Fig. 2c and d, significant differences were observed between normal and CIA mice. The abnormal expression of IL-1β and IL-17 decreased significantly with the LEF and XFHM treatments.

### Intervention in the differentiation of T, B and NK cells in CIA mice

**Fig. 3:**
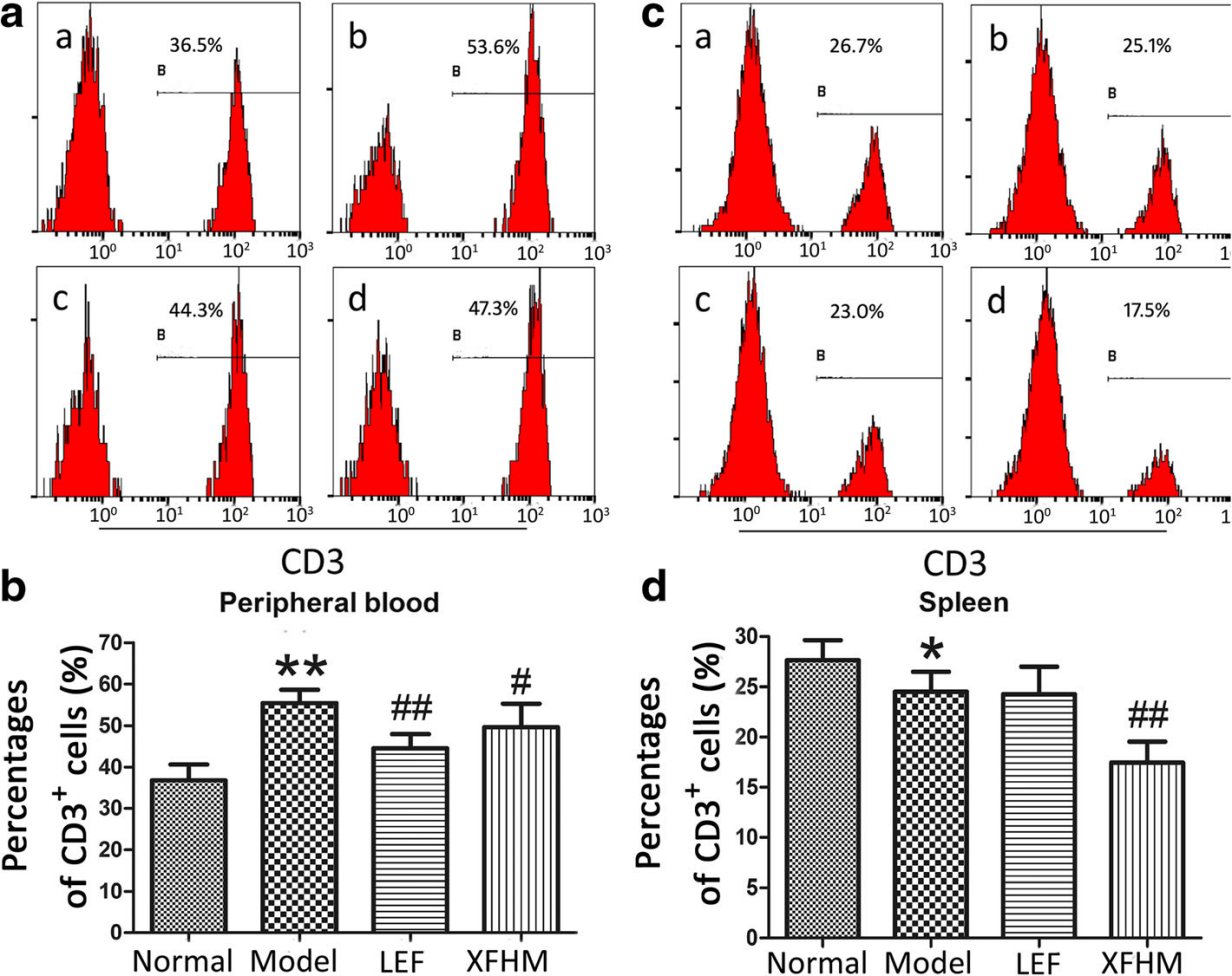
Percentages of CD3^+^ T cells in peripheral blood cells (PB) and splenocytes. **a** Histogram of flow cytometry (FCAS): **a** normal group, **b** model group, **c** LEF group and **d** XFHM group. **b** The results are presented in the bar charts. Data are presented as the means ± S.D. (*n* = 6). ^**^ *P* < 0.01 indicates model group vs. normal group; ^#^ *P* < 0.05 and ^##^ *P* < 0.01 indicate treatment groups vs. model group

**Fig. 4:**
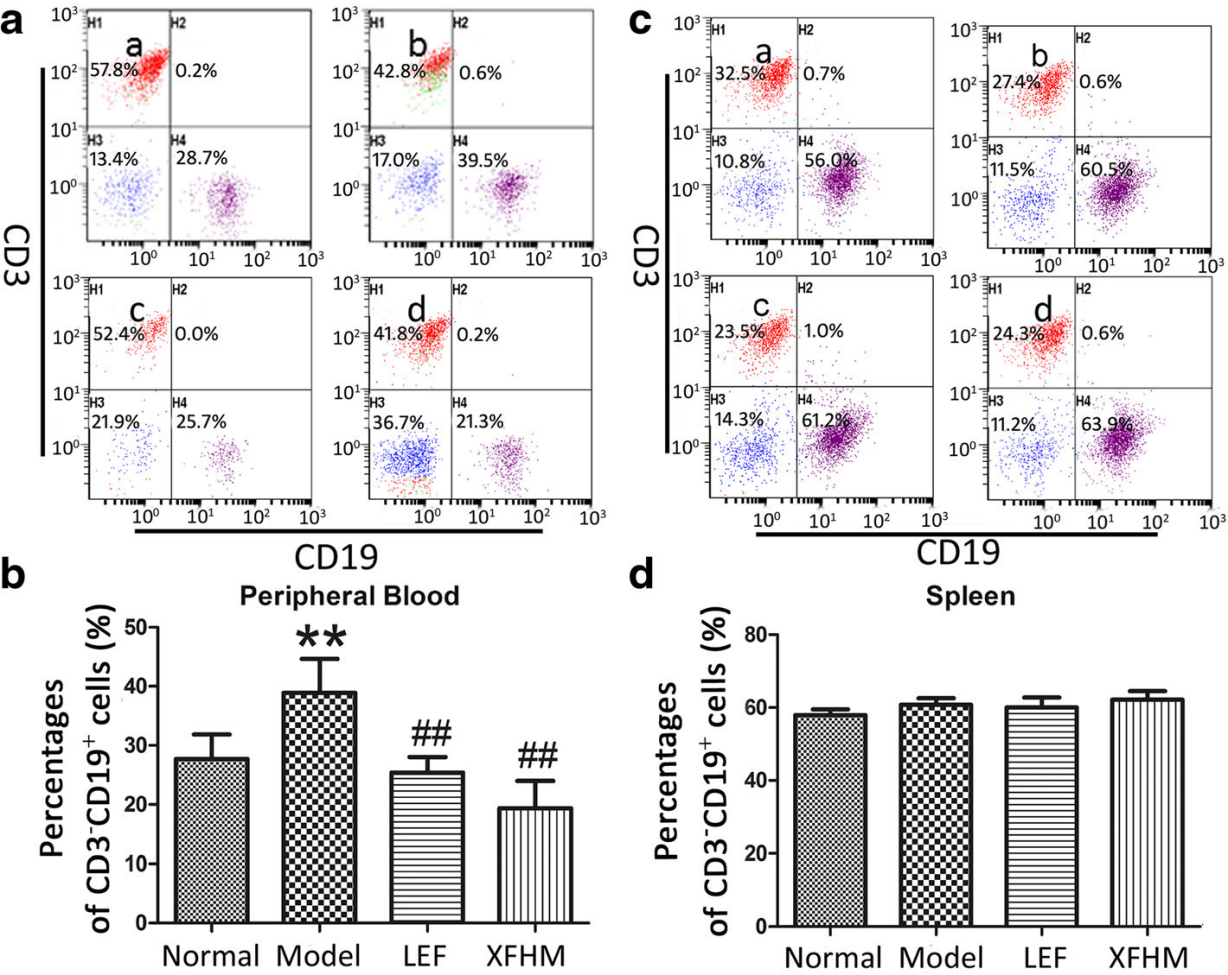
Percentages of CD3^−^CD19^+^ B cells in PB and splenocytes. **a** Histogram of FCAS: **a** normal group, **b** model group, **c** LEF group and **d** XFHM group. **b** The results are presented in the bar charts. Data are presented as the means ± S.D. (*n* = 6). ^**^ *P* < 0.01 indicates model group vs. normal group; ^##^ *P* < 0.01 indicate treatment groups vs. model group

**Fig. 5:**
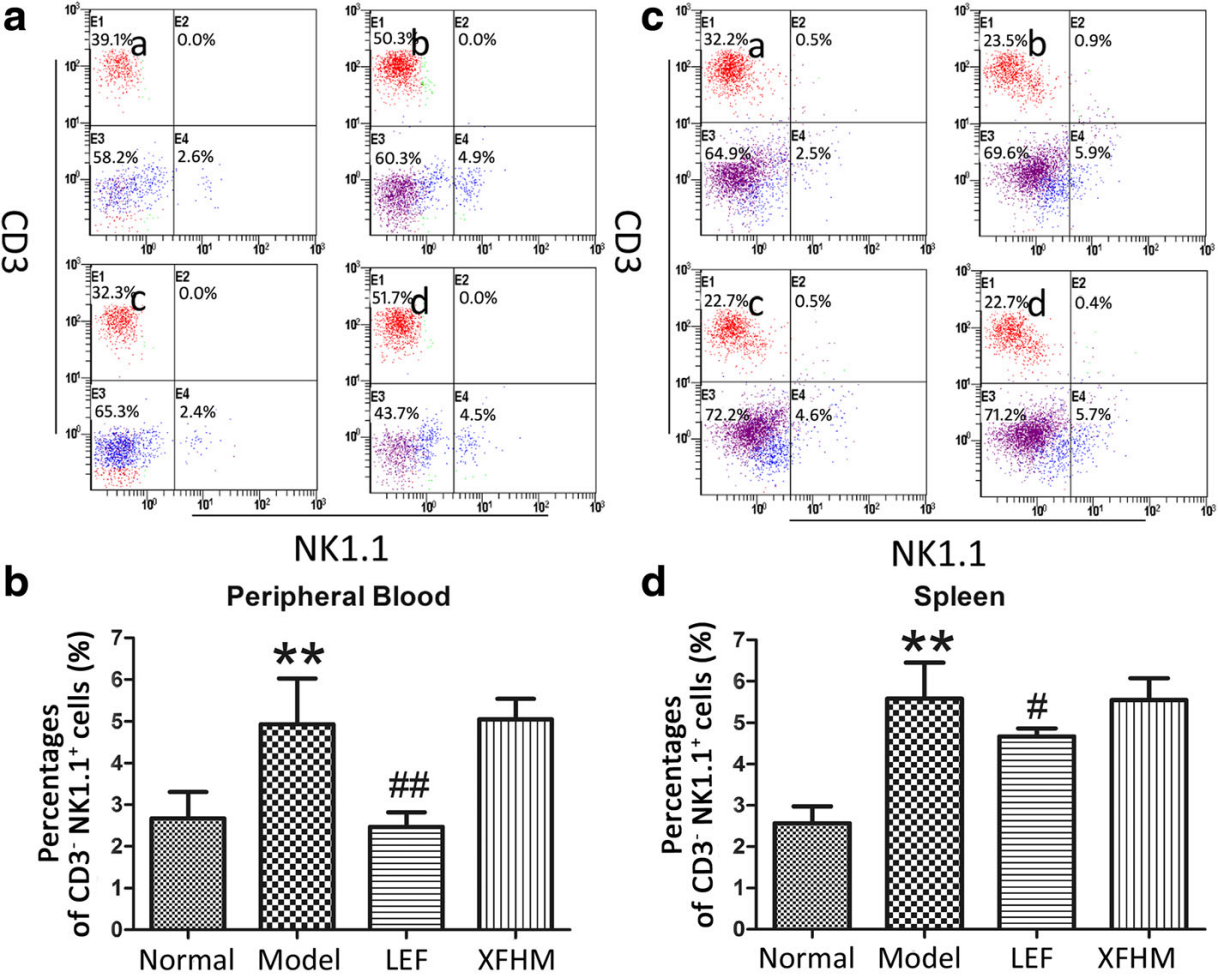
Percentages of CD3^−^NK1.1^+^ NK cells in PBMCs and splenocytes. **a** Histogram of FCAS: **a** normal group, **b** model group, **c** LEF group and **d** XFHM group. **b** The results are presented in the bar charts. Data are presented as the means ± S.D. (*n* = 6). ^**^ *P* < 0.01 indicates model group vs. normal group; ^#^ *P* < 0.05 and ^##^ *P* < 0.01 indicate treatment groups vs. model group

XFHM and LEF both could decrease the high levels of CD3^+^ T cells in PB of CIA mice (Fig. 3a and b) (*P =* 0.025 or 0.001). In addition, XFHM decreased the level of CD3^+^ T cells in spleen (Fig. 3c and d) (*P* = 0.018). The percentages of CD3^−^CD19^+^ B cells in PB (Fig. 3) and CD3^−^NK1.1^+^ NK cells in PB and spleen (Fig. 4) of CIA mice were increased significantly (*P* ≈ 0.001, 0.001 or 0.001). The abnormal rising of CD3^−^CD19^+^ B cells in PB was inhibited significantly after treatment with XFHM and LEF (Fig. 4) (*P* ≈ 0.001 or 0.001). Moreover, LEF inhibited the proliferation of CD3^−^NK1.1^+^ NK cells in PB and spleen (*P* = 0.001 or 0.029). However, XFHM had no significant regulatory effect on NK cells (Fig. 5).

### Effect of XFHM on the production of pro-inflammatory and anti-inflammatory cytokines

**Fig. 6:**
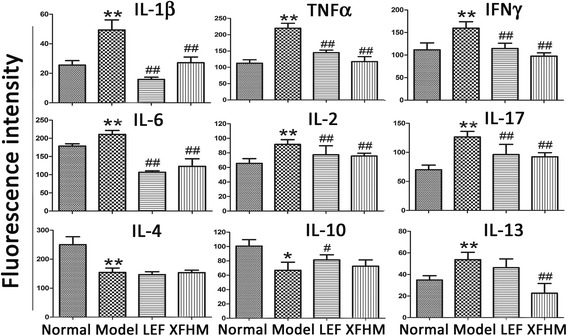
The levels of pro-inflammatory and anti-inflammatory cytokines in serum detected by inflammation antibody array assay. Data are presented as the means ± S.D. (*n* = 6). ^*^ *P* < 0.05 and ^**^ *P* < 0.01 indicate model group vs. normal group; ^#^ *P* < 0.05 and ^##^ *P* < 0.01 indicate treatment groups vs. model group

Next, we detected 40 types of pro-inflammatory cytokines in serum by Inflammation Antibody Array analysis. The results for 9 cytokines associated with RA are shown in Fig. 6. The levels of predominant pro-inflammatory cytokines contributing to the pathogenesis of RA, including IL-1β, TNFα, IFNγ, IL-6 and IL-17, were increased significantly in CIA mice. The production of those cytokines was restrained to different extent after treatment with LEF or XFHM. Amazingly, significant inhibitory effects appeared on the productions of IL-1β, TNFα, IFNγ and IL-6. However, there were no positive regulatory effects on IL-4, IL-10 or IL-13 from XFHM treatment.

### Inhibition of the activation of the NF-κB and JAK/STAT signaling pathways by XFHM treatment

**Fig. 7:**
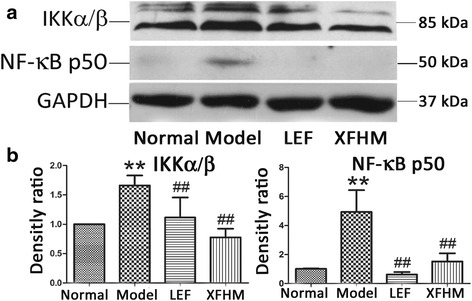
Effect of XFHM on inhibiting activation of the NF-κB signaling pathway. **a** and **b** IKKα/β and NF-κB p50 were detected in whole spleen tissue lysates by western-blot analysis. The quantified results are presented in a bar chart. GAPDH was used as an internal control. Data are presented as means ± S.D. (*n* = 6). ^**^ *P* < 0.01 indicates model group vs. normal group; ^#^ *P* < 0.01 and ^##^ *P* < 0.01 indicate treatment groups vs. model group

**Fig. 8:**
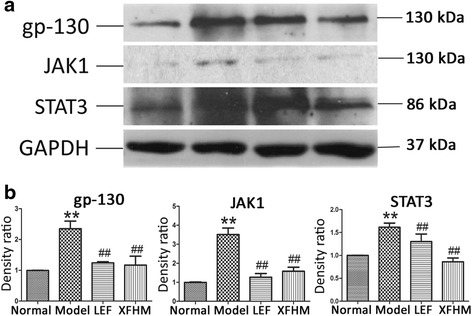
Effect of XFHM on inhibiting activation of the JAK/STAT signaling pathway. **a** and **b** gp130, JAK1 and STAT3 were detected in whole spleen tissue lysates by western-blot analysis. The quantified results are indicated by a bar chart. GAPDH was used as an internal control. GAPDH was used as an internal control. Data are presented as the means ± S.D. (*n* = 6). ^**^ *P* < 0.01 indicates model group vs. normal group; ^#^ *P* < 0.01 and ^##^ *P* < 0.01 indicate treatment groups vs. model group

We also performed western-blotting to test the production of key protein molecules of the NF-κB and JAK/STAT signaling pathways in spleen. The production of IKKα/β, NF-κB p50 (NF-κB signaling) and gp130, JAK1, and STAT3 (JAK/STAT signaling) proteins in CIA mice were increased notably, as compared with mice in the normal group (*P* ≈ 0.001, 0.008, 0.001, 0.001 or 0.001). As shown in Figs. 7 and 8, the abnormally higher levels of those molecules decreased significantly after LEF or XFHM treatment. These data suggest that XFHM exerts inhibitory effects on the production of pro-inflammatory cytokines by suppressing the activation of the NF-κB and JAK/STAT signaling pathways.

## Discussion

In TCM, combinatory therapeutic strategies according to patient symptoms are often applied to various types of diseases [30]. The combinations of herbs form multi-herbal formulae for diseases treatment. Treatment by such formulae has been verified scientifically for treatment on several diseases as an effective complementary or alternative therapy [31]. In this study, we focused on the therapeutic efficacy of a traditional herbal formula XFHM on RA, XFHM, and the mechanisms by which it regulates both the differentiation of T, B and NK cells and the production of pro-inflammatory cytokines. Suppressing effects on NF-κB and JAK/STAT signaling pathways were also detected.

As described previously, the formula of XFHM is consists of 12 medicinal herbs. It has been suggested that these herbs or the active ingredients in these herbs offer beneficial effects on RA. *Ligusticum chuanxiong Hort* has been demonstrated to have anti-inflammatory function [19]. *Radix Astragali* could reduce cell accumulation, relieve the swelling and arthritic index of joints, and down-regulate the serum concentrations of TNFα and IL-1β in adjuvant-induced arthritis [22]. *Lonicera japonica Thunb* was proved to have anti-inflammatory, anti-oxidative and anti-carcinogenic effects [21, 32]. The extract of *Radix Gentianae Macrophyllae* possesses significant antinociceptive and anti-inflammatory activities [20]. *Tail Radix Angelicae Sinensis* is characterized by hematopoietic, antioxidant, and immunoregulatory activities [23]. *Rehmannia glutinosa Libosch* and *Manis tetradactyla* were found to play effective roles in bone metabolism. *Rehmannia glutinosa Libosch* has been proved to stimulate the proliferation and activity of osteoblasts and to inhibit the generation and resorptive activity of osteoclasts [24].

The HPLC-ESI/MS^n^ analysis was used to identify the predominant chemical constituents in XFHM. Negative and positive base peak mass spectrums were measured to obtain information about their chemical constituents. The 21 chemical constituents shown in Table 1 were identified from full composition granules of XFHM. Several lines of evidence indicate that Veratric acid [33], Loganic acid [34], Paeoniflorin [35], Loganin [36] and apiin [37] have anti-inflammatory and immune-regulatory activities. Gentiopicroside [38], Astragaloside IV [39], Acteoside [40] and Prime-O-glucosylcimifugin [41] exert anti-inflammatory effects by inhibiting the activation of NF-κB and other signaling pathways. Isoimperatorin can inhibit TNFα-induced expression of VCAM-1 by up-regulating the production of PPAR-γ and translating signals to ERK1/2, PI3K, and PKC [42]. Chlorogenic acid [17] and Caffeic acid [43] have inhibitory effects on the inflammatory proliferation of synoviocytes. Wogonin can down-regulate the production of MMP-3, acting directly on articular chondrocytes [44]. Wogonoside inhibits LPS-induced angiogenesis both in vitro and in vivo [45]. Naringenin can manipulate the immunostimulatory properties of DCs and thus represents a potential therapeutic for the treatment of RA [46]. In addition, Formononetin can increase allergic responses by enhancing IL-4 production in T cells [47]. Taken together, these findings suggest that functions of the constituents of XFHM synergistically contributed to the therapeutic effects for RA treatment.

In the pathogenesis of RA, inflammatory cells including monocytes and leukocytes infiltrate into the joint tissues, along with the proliferation of synovial lining cells, then leading to pannus formation which covers the surface of articular cartilage and bone. Pannus produces various pro-inflammatory cytokines and chemokines, leading to the destruction of cartilage and bone [48]. The present results of Safranin O and immunohistochemical staining showed that XFHM could relieve the articular cartilage injury characterized by proteoglycan loss, reduced the level of pro-inflammatory cytokines IL-1β and IL-17, inhibit the inflammatory infiltration in the synovium, and thus repair the destruction of cartilage and bone.

Antigen-presenting cells present arthritis-associated antigens to T cells, thereby initiating RA pathogenesis. Activated T cells infiltrate into the synovial membrane, combine with autocrine pro-inflammatory cytokines, and then induce inflammation in the synovium. B cells, also known as antigen-presenting cells in the pathogenesis of RA, can secrete auto-antibodies, thus further stimulating the production of pro-inflammatory cytokines. The activation of T and B cells increases the production of cytokines and chemokines, contributing to a feedback loop for the interaction of T cells, macrophages and B cells [5]. NK cells have been reported to have protective and pathogenic roles in RA [49]. Interactions of NK cells with other immune cells boost the release of pro-inflammatory cytokines, contributing to inflammation of the synovium in RA. Our results indicated that XFHM can modulate the differentiation of T and B cells, inhibiting the inflammatory progress in the synovium of RA.

Within the microenvironment of inflamed articular cartilage in RA, IL-1β, TNFα, IFNγ, IL-6 and IL-17 are the dominant pro-inflammatory cytokines. These cytokines have pleiotropic effects [48]. Myeloid origin cells, including macrophages and dendritic cells, produce most of the TNFα, IL-1β and IL-6. IFNγ and IL-17 produced by T helper 1 (Th1) and Th17cells respectively, are known as the defining cytokines for these cells. These pro-inflammatory cytokines interact synergistically, participating in the proliferation and differentiation of pathogenic cells, inflammatory cell migration, pannus formation, and the process of destruction of cartilage and bone [50, 51]. IL-4, IL-10 and IL-13, as the classic anti-inflammatory cytokines, can decrease the production of inflammatory cytokines, and thus inhibit synovial inflammation and bone damage [52, 53]. In the present study, we found that XFHM decreased the production of pro-inflammatory cytokines that promote autoimmune pathology and that it displayed immunosuppressive activities. Strangely, it had no positive regulatory effect on the production of anti-inflammatory cytokines. In TCM, combinations of plant species and minerals, called formulae, are often prescribed based on clinical experience. In formulae, multiple components could interact with multiple targets and exert synergistic therapeutic efficacy. The regulatory mechanism of XFHM on the production of pro-inflammatory and anti-inflammatory cytokines needs to be researched deeply.

NF-κB, a transcription factor, is involved in inflammation, cell survival, proliferation and differentiation. Increased NF-κB activity contributes to the chronic inflammatory characteristic of RA. NF-κB protein normally exists as homo- or hetero-dimers. The homo- or hetero-dimers combine with an NF-κB inhibitor (IκB) to form a cytoplasmic complex that inhibits its entry into the nucleus. IKKα/β mediates the phosphorylation of IκB. IκB phosphorylation prompts its proteasome-mediated degradation through ubiquitination. NF-κB dimers are released and free to migrate into the nucleus. The released NF-κB dimers bind to promoter regions and induce the expression of target genes. Previous investigations have detected NF-κB activation in RA synovium, and the expression of p50 and p65 has been observed in synovial intimal lining cells by immunohistochemical analysis [54, 55]. Furthermore, the JAK/STAT signaling pathway, known as the IL-6 pathway, offers novel potential therapeutic opportunities that aim to prevent bone destruction in RA [56]. IL-6 activates the JAK/STAT signaling cascade through binding to gp130, which triggers the phosphorylation of JAK/STAT in primary rheumatoid synoviocytes. Phosphorylated STAT3 proteins are dimerized, consequently translocating into the nucleus, and then regulate target gene transcription [57]. In this study, we discovered that XFHM could regulate the secretion of pro-inflammatory cytokines by inhibiting the activation of the NF-κB and JAK/STAT signaling pathway induced by TNFα, IL-1β or IL-6.

LEF is a classic DMARD for RA treatment. It inhibits the enzymatic activity of dihydroorotate dehydrogenase and has anti-proliferative effects. The activation of T cells depends on signal transduction of the dihydroorotate dehydrogenase pathway particularly. LEF also inhibits the activity of pro-inflammatory cytokines such as TNF and IL-1, relieves inflammation, delays the progress of joint and cartilage destruction, and thus improves the quality of life of RA patients [58]. In this study, we also obtained significant anti-inflammatory effects and inhibitory action on bone destruction through LEF treatment for CIA. However, a recent study indicated that LEF had myelosuppressive and hepatotoxic potential in a rat model of RA [59]. Adverse drug reactions to LEF, such as pruritus, loss of appetite, weak, dizziness, diarrhea and erythra, also occur frequently in clinics. Multi-herbal formulas based on traditional medicine have been scientifically verified as a complementary and alternative therapy for the treatment of various diseases. Formulae composed of a mixture of natural products have the presumed ability to target multiple sites. In our study, XFHM was verified as an effective medicine for RA treatment. It can also avoid the myelosuppressive, hepatotoxic potential and other adverse reactions that occur due to chemical drugs including LEF. In the present study, the results indicated that the effects of anti-inflammation and inhibition of bone and cartilage destruction of XFHM were equal to or exceeded those of LEF.

## Conclusions

In summary, our results indicate that XFHM can suppress the inflammatory swelling in joints, inhibit inflammatory infiltration in synovium and relieve cartilage and bone destruction in CIA mice. The main mechanisms likely involve to the modulation of T, B and NK cell differentiation, a decrease in inflammatory hyperplasia of synovium, and pro-inflammatory cytokine secretion by suppressing the activation of NF-κB and the JAK/STAT signaling pathway. The use of XFHM should be an optional complementary and alternative therapy for RA treatment.

## Additional file

Additional file 1: Figure S1. The infrared spectrum fingerprint (IRFP) of YQHXJD compound. The x-axis indicated wavelength of absorption, and y-axis indicated absorption intensity. (PDF 128 kb)

## Abbreviations

CFA: Freund’s complete adjuvant
CIA: Collagen-induced arthritis
DAD: Diode array detector
DMARD: Disease-modifying anti-rheumatic drug
FACS: Flow cytometry
GAPDH: Glyceraldehyde 3 - phosphate dehydrogenase
HPLC-ESI/MS^n^: High performance liquid chromatography-electrospray ionization/mass spectrometer
IFA: Incomplete Freund’s adjuvant
IFN: Interferon
IFRP: Infrared spectrum fingerprint
IL: Interleukin
JAK: Janus kinase
LEF: Leflunomide
NF-κB: Nuclear factor κB
NK: Natural killer
RA: Rheumatoid arthritis
S.D.: Means ± standard deviation
SDS-PAGE: Sodium dodecyl sulfate-polyacrylamide gel electrophoresis
STAT: Signal transducers and activators of transcription
TCM: Traditional Chinese medical
TNF: Tumor necrosis factor
XFHM: Xian-Fang-Huo-Ming-Yin
