# Supplementary material for: Herbal formula Xian-Fang-Huo-Ming-Yin regulates differentiation of lymphocytes and production of pro-inflammatory cytokines in collagen-induced arthritis mice
Source: BMC Complement Altern Med. 2017 Jan 5;17:12. doi: 10.1186/s12906-016-1526-x (PMC5216578; doi:10.1186/s12906-016-1526-x)
Supplement: Additional file 1: Figure S1. — The infrared spectrum fingerprint (IRFP) of YQHXJD compound. The x-axis indicated wavelength of absorption, and y-axis indicated absorption intensity. (PDF 128 kb) [file 12906_2016_1526_MOESM1_ESM.pdf]

Supplementary material

The infrared spectrum fingerprint (IRFP) of the FCG of YQHXJD granule

Fourier-transform infrared spectroscopy is an alternative identification technique which is considered to replace traditional method such as high performance liquid chromatography fingerprint analysis for evaluating the quality of traditional Chinese medicine (TCM) (Sun et al. 2014). IRFP presents wide absorption band, less absorption peaks, poor characteristic and decreased specificity for denoting the total superposition of various chemical composition of the atoms and groups in TCM and herbal drugs (Wu et al. 2008, Xu et al. 2013). We used IRFP in this study for revealing the complex system of YQHXJD granule. As shown in Figure S1, we used technique of computer aided analysis and mathematical methods for displaying the chemical characteristics of this FCG, according to the spectral data points from 4,000-400 cm^{-1}. Several characters of YQHXJD granule were shown in the IRFP, such as the strongest peaks in the range of 860.96-1243.32 cm^{-1} mainly attributed to the stretching vibration of C-O, which displayed the characteristic absorption of glycosides and terpene lactones, the stronger peak at 3339.14 cm^{-1} belonging to stretching vibration of O-H groups in associated phenolic hydroxyl of flavonoids and phenoliacids, the peaks at 2933.83 and 1414.40 cm^{-1} assigning to the stretching vibration of –CH2 and –CH3 groups corresponding to alkanes and alkenes, and the peaks in the range of 707.82-439.24 cm^{-1} attributed to the stretching vibration of C-X pertained to the characteristic absorptions of chlorides and bromides (Figure S1).

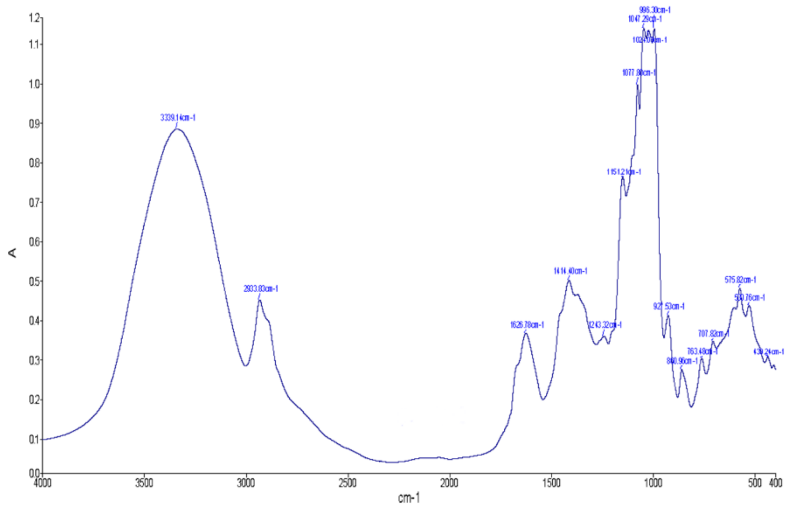

Figure S1. The infrared spectrum fingerprint (IRFP) of YQHXJD compound. The x-axis indicated wavelength of absorption, and y-axis indicated absorption intensity.
